# Forest bat population dynamics over 14 years at a climate refuge: Effects of timber harvesting and weather extremes

**DOI:** 10.1371/journal.pone.0191471

**Published:** 2018-02-14

**Authors:** Bradley S. Law, Mark Chidel, Peter R. Law

**Affiliations:** 1 Forest Science Unit, NSW Primary Industries, Parramatta, Sydney NSW, Australia; 2 Centre for African Conservation Ecology, Department of Zoology, Nelson Mandela Metropolitan University, Port Elizabeth, South Africa; Università degli Studi di Napoli Federico II, ITALY

## Abstract

Long-term data are needed to explore the interaction of weather extremes with habitat alteration; in particular, can ‘refugia’ buffer population dynamics against climate change and are they robust to disturbances such as timber harvesting. Because forest bats are good indicators of ecosystem health, we used 14 years (1999–2012) of mark-recapture data from a suite of small tree-hollow roosting bats to estimate survival, abundance and body condition in harvested and unharvested forest and over extreme El Niño and La Niña weather events in southeastern Australia. Trapping was replicated within an experimental forest, located in a climate refuge, with different timber harvesting treatments. We trapped foraging bats and banded 3043 with a 32% retrap rate. Mark-recapture analyses allowed for dependence of survival on time, species, sex, logging treatment and for transients. A large portion of the population remained resident, with a maximum time to recapture of nine years. The effect of logging history (unlogged vs 16–30 years post-logging regrowth) on apparent survival was minor and species specific, with no detectable effect for two species, a positive effect for one and negative for the other. There was no effect of logging history on abundance or body condition for any of these species. Apparent survival of residents was not strongly influenced by weather variation (except for the smallest species), unlike previous studies outside of refugia. Despite annual variation in abundance and body condition across the 14 years of the study, no relationship with extreme weather was evident. The location of our study area in a climate refuge potentially buffered bat population dynamics from extreme weather. These results support the value of climate refugia in mitigating climate change impacts, though the lack of an external control highlights the need for further studies on the functioning of climate refugia. Relatively stable population dynamics were not compromised by timber harvesting, suggesting ecologically sustainable harvesting may be compatible with climate refugia.

## Introduction

Many species face challenges of climate change interacting with habitat alteration. Understanding how these perturbations affect life history attributes and drive population dynamics is vital for improving future conservation and management of species. Long-term data sets are needed to quantify variation in population dynamics, especially in relation to management interventions and global-climate-change induced weather extremes [1, 2, 3]. The possibility that certain environments can resist the effects of climate change more than others has raised the notion of ‘climate refugia’ as important components of management plans to mitigate climate change [4]. Some progress has been made in identifying drought refuges in arid environments [5], but existing proposals for evaluating the value of refugia often rely on mechanistic model frameworks [6]. In particular, buffering of population dynamics in potential climate refugia during weather extremes is relatively underexplored.

Moist, montane forests offer the potential to provide climate refugia [7], but are also often subject to timber harvesting and little is known about how such disturbances might compromise this potential. For instance, it is poorly known whether disturbed forests function as ecological traps or sinks [8, 9, 10], which would undermine their value as possible climate refugia. Investigation of these issues requires suitable study species. Insect-eating bats are diverse and functionally important taxa, playing key roles in suppressing insects [11]. Considered sensitive to disturbance, bats are often proposed as indicator species of environmental health [12]. As such, forest bats in particular, are promising taxa for exploring the potential of forests as climate refuges and the possible adverse effects of timber harvesting on that potential.

Although the broad life-history traits of bats are well known, with some unusually long-lived (20–30 years) yet with low fecundity [13, 14, 15], long-term studies, vital for understanding environmental changes, are particularly rare for bats, especially for dynamic processes pervading long-lived systems like forests [16]. Most studies of bat population dynamics have focused on colonial bats roosting in subterranean or artificial structures [3, 17, 18, 19, 20] and their survival is sensitive to introduced predators [21] and climate, including hot dry summers [3], warm winters [21, 22], and excessive rainfall [23]. Few studies exist for forest bats using natural roosts in tree hollows, where only a small proportion of the population may be detected [21, 24].

While a significant amount of research has investigated the response of bats to timber harvesting, changes in population dynamics in relation to forest management are scarce [16]. Short-term studies have demonstrated that dense clutter in regrowth forests impedes efficient foraging by many bat species, resulting in low activity [25. 26, 27, 28]. While a range of studies demonstrate that flyways along tracks in dense regrowth forest support high activity of bats comparable to unlogged forest [25, 27, 28, 29], tracks represent a small proportional area of the landscape (~ 3%) in timber production forests (e.g. [28]), so it is unclear to what extent tracks mitigate the effects of extensive cluttered regrowth at the scale of a local population.

Our goal was to collect fundamental demographic data for several species of forest bats in a setting that would also test the value of their forest habitat as a climate refuge and the robustness of that value after timber harvesting. We used 14 years (1999–2012) of mark-recapture data from a suite of small tree-hollow-roosting vespertilionid bats at replicated sites within a long-term experimental forest in the Barrington Tops area of north-east New South Wales, Australia. In connection with logging disturbance, we aimed to test the collective effectiveness of a range of environmental mitigation measures (e.g. retention of riparian buffers, presence of tracks, etc) by comparing survival, abundance and individual body condition between regrowth and unlogged. The experimental forest supports small catchments of unlogged forest and forest regenerating from timber harvesting in 1983, when ecological forest management was in its early stages. Bats in this study area maintained high activity on tracks in 16 year old regrowth, but off-track activity was negligible [25].

Our study also spanned and extended beyond a period of low rainfall known in Australia as the ‘Millennium drought’ (1997–2010), which was unprecedented in the recorded history of southern Australia [30]. Many species declined during this period, including the koala *Phascolarctos cinereus* [31], woodland birds [32] and insectivorous marsupials [33, 34]. How such species respond in climate refugia is generally unknown. The high elevation, topographically complex, mixed *Eucalyptus* and rainforests of our study area are considered climate refugia [35, 36]. Given the unplanned nature of this drought, our secondary study aim was to use this opportunity for a retrospective assessment of the effect of extreme weather events in a purported climate refuge, overlayed with two contrasting timber harvesting histories. We acknowledge the absence of a planned control outside our study site, but contend studies such as ours remain useful because logistical difficulties make longitudinal studies of population dynamics at multiple sites a rarity.

Specific predictions of our study were: (i) if logging impacts bat populations, then survival rates, abundance and body condition in unlogged catchments should be greater than in regrowth catchments irrespective of weather. (ii) if climate refugia buffer against extreme climatic events, we expected to see resilience in bats; i.e. weak association between survival and abundance with climate co-variates and good body condition of residents. (iii) if logging disturbance compromised the study site as a climate refuge, bats should show lower survival, body condition, and/or abundance in logged treatments especially during the period of more extreme weather.

## Materials and methods

### Study area

The study area was located in an experimental section (450–940 m elevation) of Chichester State Forest, 200 km north of Sydney, Australia. The experimental section, which comprises eight small (13–97 ha) catchments, was established in 1974/75 to investigate the effects of logging on water flow and quality (see [37] for site description), embedded within extensive forests at varying stages of post-logging regeneration. The site average rainfall from 1974–2013 was high (1547 mm), and despite five consecutive years of below average rainfall during the study, only 2002 recorded < 1000 mm.

Six of the eight catchments were clearfelled in 1983, with the remaining two catchments of old growth left undisturbed [37]. Unlogged buffers of various widths lined creeks (20 m minimum each side of creek). Prior to experimental logging, tall wet sclerophyll forest (> 35 m) covered most of the catchments, dominated by Sydney blue gum *Eucalyptus saligna*, silvertop stringybark *E*. *laevopinea* and rainforest. Rainforest species dominated creek-lines and much of the understorey. In 1997, after 14 years of regrowth, trees occurred at a much greater density in the regrowth catchments (3500–6100 stems ha^-1^) compared to unlogged catchments (744 stems ha^-1^) [37]. Rainforest understorey contributed most stems (700 ha^-1^) in the unlogged catchments, with overstorey eucalypts occurring at a density of just 44 ha^-1^.

### Experimental design

**Fig 1:**
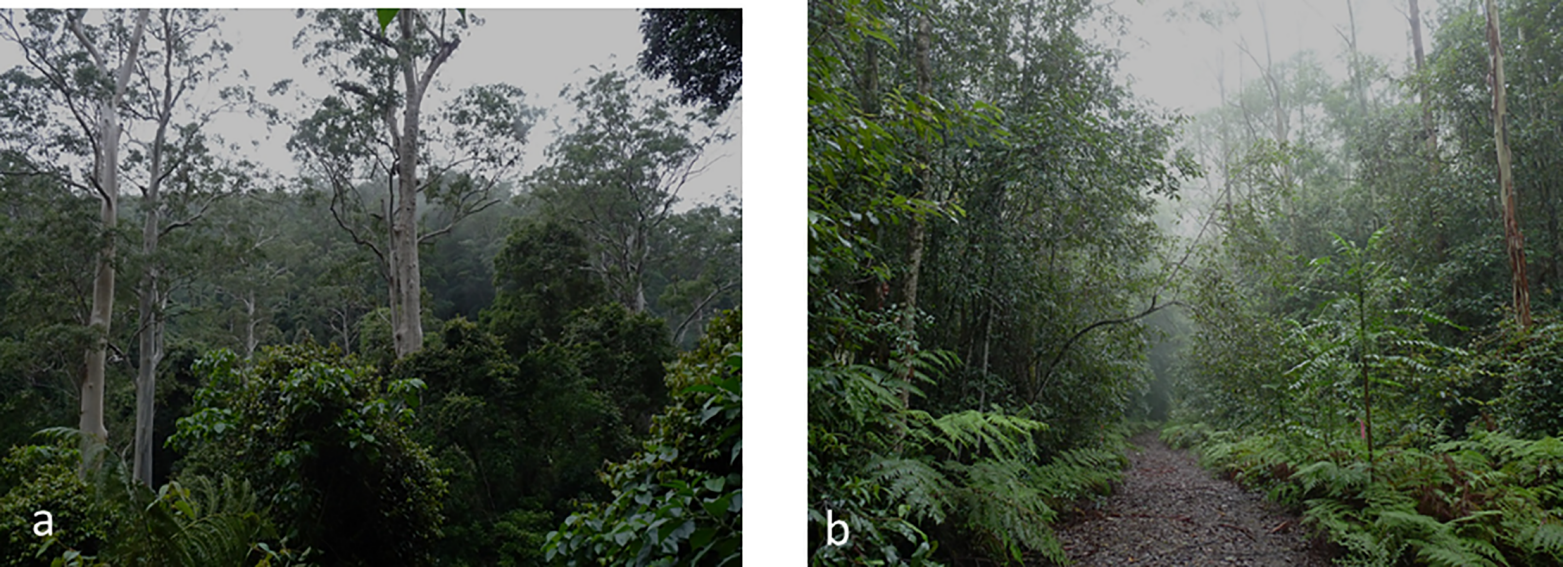
High elevation eucalypt forest in the climate refuge study area. (a) unlogged forest catchment showing tall emergent eucalypts above a dense rainforest understorey (b) 27 year old regrowth catchment showing dense stands of mixed eucalypts and rainforest species with a small 4WD trail used for harp-trapping bats.

Because of the area requirements of bats, each site replicate spanned two adjacent catchments of the same forest treatment, extending the small spatial scale of the individual catchments in the original hydrology experiment [37] to an average of 78 ha per treatment. Unlogged treatments were dominated by old growth forest (includes rainforest) (a mean of 68% of a 500 m circular buffer centred on unlogged treatments) while eucalypt regrowth (excludes riparian buffers) represented an average of 56% of the buffer surrounding regrowth treatments. We compared bat population dynamics in four areas of regrowth forest with two areas of unlogged forest (Fig 1). One regrowth area and unlogged area sampled lower elevation forest, one regrowth area sampled mid-elevation, while two regrowth and one unlogged area sampled higher elevations. Three different clearfell logging practices yielded the regrowth forest: logging plus a regeneration burn at Corkwood, logging without a regeneration burn at Raingauge 9, and logging followed by eucalypt plantation establishment at Kokata and Coachwood. Riparian buffers were retained on creeks and scattered old, hollow trees and unharvested rainforest were also retained. Although logging practices varied, vegetation structure in logged treatments was similar, based on estimates of stem density and vegetation cover, after 16 years of regrowth when our study began [25, 37].

### Bat sampling

The six study locations were sampled every year in autumn (~ four months after parturition) for 14 years (1999–2012). At each site, we standardised trapping effort, with each capture event consisting of two consecutive nights of trapping using 3 harp-traps set within a 200 m length of a 4WD trail. Time of year was standardised to the same two week period at the beginning of autumn (March) each year. We conducted one trapping event per year to minimise trap avoidance behaviour, except the first two years of the study when we employed three trapping events each autumn (separated by ~ two weeks) for the purpose of maximising the number of bands in the population for recapture in later years. Also, we did not extend trapping beyond two nights for each site to minimise trap avoidance. The same locations were trapped each year of the study.

We applied approved flanged-metal bat bands from the Australian Bird and Bat Banding Scheme to the forearm of captured bats. Study protocols were approved by the Department of Industry-Lands and Forestry, Forestry Corporation animal ethics committee (Animal Research Authority: 12/17). Band injuries on recaptured bats were noted (0 = no injury, 1 = minor abrasion, 2 = minor swelling, 3 = serious swelling of forearm requiring band removal). We recorded < 1% of recaptures with a swelling that required us to shift the band to the other forearm, indicating that band injuries were rare (unlike split metal types, [38]). Bats were sexed, weighed, forearm lengths measured and teeth wear noted as an independent, though coarse, method of aging bats (1 = shiny and sharp, 2 = dull, but sharp, 3 = dull and noticeably worn). We note that teeth wear will vary based on a species’ diet, but the species we studied have a mixed diet of small moths and beetles. As epiphyses had typically fused by the time of Autumn trapping, we were unable to reliably identify juveniles by this method. Eight percent of bats were classified as juveniles (3–4 months old) at capture, but on some occasions none were identified as juveniles.

### Modelling bat recaptures

All 18 capture events, three in each of 1999 and 2000 and one per year thereafter, were treated as distinct capture events in the capture-mark-recapture (CMR) analyses. Within-year capture events in each of 1999 and 2000 were two weeks or a month apart, while successive capture events from 2001–2012 were one year apart.

We employed program MARK to construct Cormack-Jolly-Seber (CJS) models for apparent survival and recapture with time, species, sex, and logging treatment (regrowth versus unlogged) as groups or effects and a two-week time step. We then used the Akaike information criterion (AIC_c_) [39] for model comparison and selection. This approach provides a systematic method to select parsimonious models, as measured by the number of parameters in the model, which trade some bias for precision in parameter estimates. We reduced model complexity in order to capture the strongest biological signals in the data and avoid being misled by poorly estimated parameters [40]. One outcome of this approach was that site elevation could not be incorporated into our model because of the additional complexity it would have added given the number of sites and bats sampled and the need to allow for the presence of transients in the modelling. Nevertheless, by having logging treatment represented at both high and low elevation, we avoided confounding logging treatment with an elevation effect.

Because we could not reliably discriminate adults from juveniles when banding in autumn we did not distinguish age classes and this heterogeneity may have introduced some bias into our survival estimates. However, most males (81%) had swollen and descended testes at this time of the year and so were functionally adult. Bats are highly mobile, suggesting that models distinguishing between transients and residents might be relevant, especially as some transients may be sub-adults.

We used program U-CARE [41] for goodness-of-fit testing, which indicated the presence of transients (i.e., bats with zero apparent survival) but provided no evidence for a trap effect or overdispersion requiring the use of QAIC when species and time were included as effects in the global model (see Supplement for details). We therefore employed AIC_c_ for model ranking and utilized the time-since-marking (TSM) model of MARK [42], §7.4) to distinguish between newly marked (mixture of transients and residents) and already marked bats to implement the model of Pradel *et al*. (1997) [43]. For such models, survival of already marked bats is interpreted as survival of residents. The survival of newly marked bats is used to compute the proportion of newly marked bats that are residents (as the ratio of survival of newly marked bats to that of already marked bats). Note that as recapture probability is conditioned on the presence of marked individuals, it is unaffected by the presence of transients.

The TSM model with time*species*sex*treatment dependence served as our global model but the very large number of model parameters and the typical sparseness of mark-recapture data entailed it served primarily as a heuristic model. The CJS models and AIC_c_ comparisons led us to choose the structure sex*time for recapture (no evidence for logging treatment or species effects) but we initially retained time*species*sex*treatment for survival in the TSM model. While maintaining a distinction in survival between newly and already marked bats and between capture events within years and capture events between successive years, we simplified the dependence of survival on effects iteratively until we obtained models for which MARK estimated all remaining parameters. Some simplifications reflected insufficient data to discriminate effects, e.g., to discern effects on survival between capture events within years. Time dependence remaining in models was simplified by modelling it with logistic regression using the weather covariates described in Table 1. We hypothesized that survival over years might covary with over-winter conditions, as measured by MinTempWint when bats are torpid [21, 22]. We also conjectured that spring-summer conditions, both temperature and rainfall, prior to capture when bats are active and foraging would be important, e.g., through influences on insect abundance [3] for both survival and recapture, while temperature and rainfall during the capture period might influence recapture (Table 1). Note that 6laggedRain was negatively correlated (-0.8) with MaxTempSumm so these covariates were never employed in the same model. The same value of MaxTempSumm was employed for capture events within the same year. In a preliminary analysis, we also tested the mean maximum daily temperature during the previous winter, mean minimum daily temperature in the previous summer, rainfall in the previous 12 months (definitions analogous to those in Table 1), rainfall during the capture period and 6- and 12-month lagged measures of Southern Oscillation Index, but none of these covariates proved competitive with those listed in Table 1 and were discarded.

**Table 1:** Covariates employed in modelling time dependence in survival and recapture.

Covariate	Definition
MinTempWint	the mean minimum daily temperature in preceding autumn-winter (March-August)
MaxTempSumm	the mean maximum daily temperature in preceding spring-summer (September–February)
6laggedRain	the total rainfall over the 6 months prior to capture
AvMinTempDur	the mean minimum daily temperature during the capture period

This procedure for simplifying both group structure and time dependence yielded a model singled out by AIC_c_ ranking, i.e., which was more competitive than more complex models or similarly complex models with alternate structure. The recapture structure was p(sex+ MaxTempSumm + AvMinTempDur) and the survival structure denoted here φ(1) is described in Table 2. This model was further simplified by reducing dependencies of covariates on group effects, and/or removing group effects or covariates, resulting in a collection of 35 models over which AIC_c_ increased gradually. Thus, many slight variations of the model φ(1)p(sex+ MaxTempSumm + AvMinTempDur) structure were competitive alternatives (S2 Table). We adopted the AIC-cut-off notion with a value of four for a confidence set of models [39]:170). Throughout our analysis we exploited Burnham and Anderson’s [39] parsimony argument to reject as competitive a model that resulted by the addition of one parameter to a model already under consideration without changing the deviance.

**Table 2:** Survival structure in model φ(1)p(sex+ MaxTempSumm + AvMinTempDur). The two-letter species abbreviation indicate survival rates for that species, qualified if the rate was sex specific by adding M or F and treatment specific by adding R(regrowth) or U (unlogged), e.g., CmFU, designates a survival rate for female *Chalinolobus morio* in unlogged treatments. The absence of a qualifier means the survival rate is independent of that effect. Coincidences between survival rates are expressed with equality signs. All the survival parameters in the second column were modelled as logistic regressions on the covariate MaxTempSumm with the same regression coefficient but their own intercept, independently of whether they were newly or already marked. Note that survival rates *within the same year* only apply to the capture season, and should not be extrapolated beyond this period.

Survival parameters	Constants	Vary inversely with MaxTempSumm	Vary positively with each of MinTempWint and 6laggedRain
*Newly* marked bats: between successive capture events *within* the same year	Cm = Vd = Vp = VrR; VrU		
*Already* marked bats: between successive capture events *within* the same year	Cm = Vd = Vp = Vr		
*Newly* marked bats: *between* successive capture events in *different* years	VpM	Cm = Vd = VrR; VpFU; VrU	VpFR
*Already* marked bats: *between* successive capture events in *different* years	Cm = Vd = VrR; VpFR	VpM; VpFU; VrU	

Our final estimates of survival and recapture were obtained by model averaging over the confidence set [39], a compromise between accommodating the lack of a clear-cut model structure and excluding poor models from contributing to parameter estimation [44]. Standard errors for parameters derived from MARK output (e.g., survival rates converted to annual time steps, proportion of residents) were estimated using the delta method.

### Abundance (population size)

Modelled recapture probability is the probability of capturing a marked individual present at the capture event. For abundance estimation, we assume that, apart from the influence of modelled effects on recapture, all bats present at the capture event, whether marked or unmarked, transient or resident, were equally likely to be trapped, i.e., we assume that the modelled recapture probability also estimates the probability of capture of any bats present at the capture event. For each sex of each species, at each site, abundance of bats at a capture event was estimated by dividing the number caught at that event by the relevant probability of capture at that event, and the SE of this estimate was estimated by the delta method ([45], equations (18.6) and (18.23)). To estimate the number of residents at a capture event, the numbers of already and newly marked bats caught were each divided by the recapture probability to estimate their separate numbers at the capture event. The estimated number of newly marked bats was multiplied by the estimate of the proportion of residents amongst the newly marked bats and this estimate added to the estimate of already marked bats to obtain the overall estimate of the number of residents at the capture event (and SEs again obtained by the delta method).

### Mean life expectancy

Mean life expectancy after banding was calculated from survival estimates for resident bats of different species, all of which were banded in autumn when they were at least three months old. We used the formula of Seber (1982) [46], though note that use of apparent survival will underestimate life expectancy:

lifeexpectancy=−1/ln(annualsurvivalprobability)

### Analysis of logging effects: Abundance and body condition

For abundance, we observed general patterns relating to sex, species, and treatment (site) by averaging over capture events. To test whether logging history influenced estimated bat abundance at a site, we summed estimated male and female abundance separately for each species in each year and then averaged across years per site. We used pre-planned contrasts to compare between logging treatments using trap-site elevation as a co-variate. Body condition was calculated for each individual by dividing body mass by forearm length. Mass was measured with a Pesola spring balance (50 g) and forearm was measured with a Vernier caliper. All measurements were standardised by handling bats in the afternoon, after retrieval from harp traps in the early morning. However, we acknowledge that different individuals would have foraged for different periods prior to capture on the previous night and this may have led to additional variation in body condition measures. Body condition was used as the response variable in a mixed model analysis with logging treatments, species and sex and all interactions as fixed effects and individuals as a random factor. A significant interaction with logging was taken as evidence for a logging effect. Only recaptures were analysed as these were known residents that experienced conditions that could be confidently allocated to a treatment.

### Analysis of time series: Abundance and body condition

To examine trends in abundance over time, for each capture event, we summed over sex and species obtaining a vector of six site-specific time series and also summed over sex and sites to obtain a vector of four species–specific time series. Dynamic factor analysis (DFA; [47]) expresses a vector of time series as a linear combination of random walks and covariates (here MaxTempSumm) plus residual, and thus searches for common trends in multiple time series. DFA was implemented in package MARSS in R [48]. We used the AIC_c_ output of MARSS to select amongst simplifications of each global model. A DFA was also conducted to assess trends in body condition over time with species and sex kept separate, but summed over sites, to give a vector of eight time series.

## Results

Fourteen years of trapping led to 3043 trap records, 32% of which were retraps of banded bats. Sufficient data for modelling survival and abundance was available for four species that varied in body mass: *C*. *morio* (8 g), *V*. *darlingtoni* (6 g), *V*. *regulus* (5 g) and *V*. *pumilus* (4 g). For these four species, there were 2061 releases of 1532 distinct individuals (Table 3). A high percentage of bats in each species was only caught once (*Chalinolobus morio* 79%, *Vespadelus darlingtoni* 72%, *V*. *pumilus* 80%, *V*. *regulus* 72%).

**Table 3:** Capture frequency and counts of individual bats per species (18 of these captures occurred in 2012, the final capture event, and eight marked bats were not re-released (deaths) at earlier capture events, so of 2087 captures, 26 did not constitute releases).

Capture frequency	*Chalinolobus morio*	*Vespadelus darlingtoni*	*Vespadelus regulus*	*Vespadelus pumilus*
1	195	519	165	262
2	35	155	44	45
3	15	37	10	14
4	2	8	8	6
5	0	3	3	2
6	0	3	1	0
Totals	247	725	231	329

### Survival model structure

Initial AIC_c_ ranking of TSM models eliminated more complex models in favour of a model denoted φ(1)p(sex+ MaxTempSumm + AvMinTempDur). We describe the structure of this model in detail as final model selection is conveniently described in terms of variations on this particular model structure. The survival component φ(1) is given in Table 2 while recapture was modelled as a logistic regression on the two covariates MaxTempSumm and AvMinTempDur (Table 1 and S1 Table). For each of these two covariates, the intercept, but not the slope, depended on sex; i.e., each covariate had an additive structure with respect to the interaction with sex, and the slope was positive.

Of the 35 models obtained by further simplifying the model φ(1)p(sex+ MaxTempSumm + AvMinTempDur), 22 fell within four AIC_c_ units of the AIC_c_-best-ranked model, including the model φ(1)p(sex+ MaxTempSumm + AvMinTempDur) itself at 2.5 ΔAIC_c_ units. These 22 were selected as the models over which survival and recapture parameters were averaged (S2 Table). Since the models that we averaged were simplifications of the model structure in Table 2, we employed that structure to describe the model-averaged survival and recapture parameters.

### Variation in recapture probability

**Fig 2:**
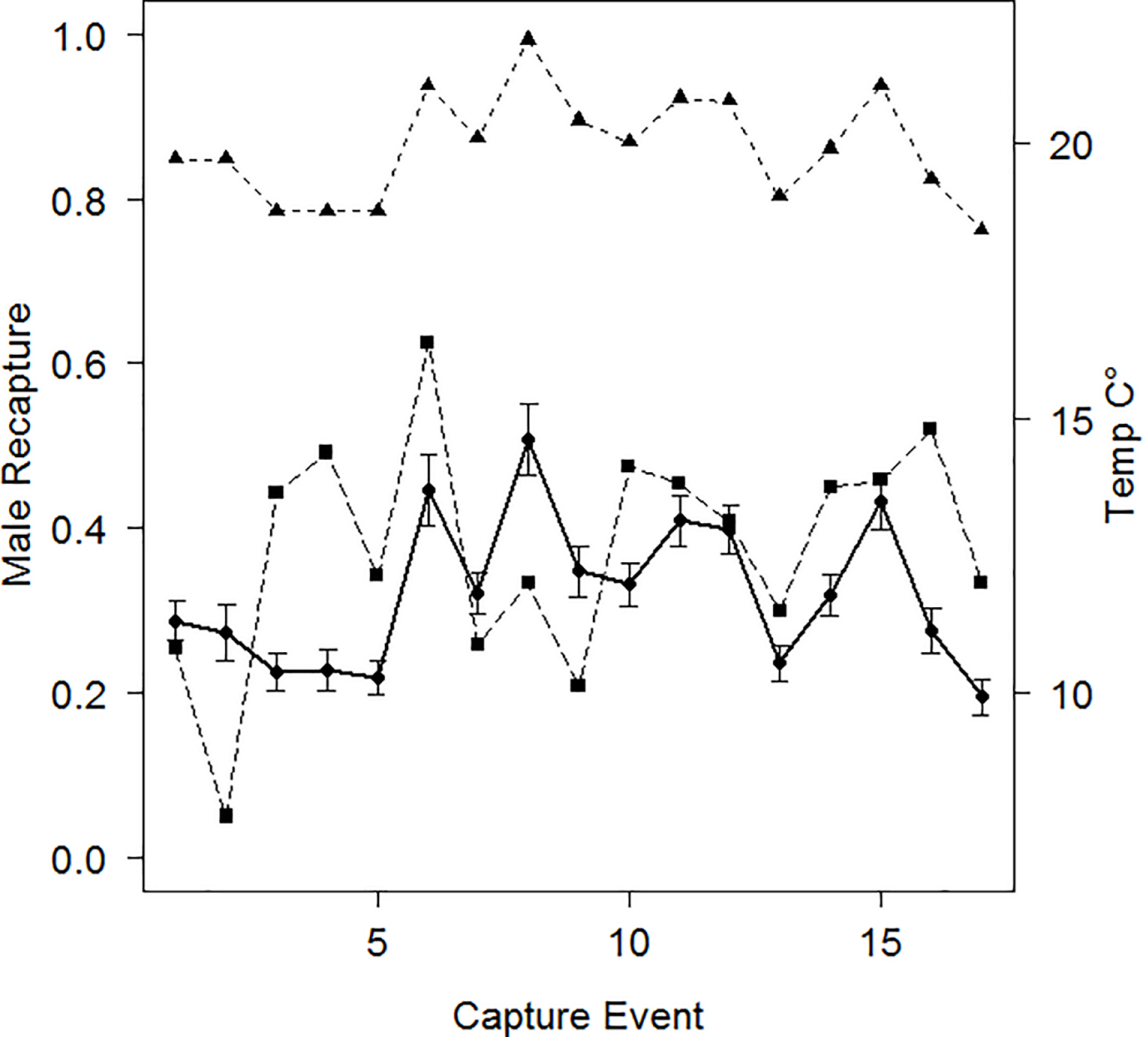
Male recapture probability and its covariates. Male recapture probability (●) at the *n*’th capture event (*n* = 1 is 99_2, the second capture event in 1999; *n* = 2, 99_3, the third capture event in 1999; *n* = 3, 4, and 5, the first, second, and third capture events in 2000, respectively; and *n* > 5 is the capture event in year 2000 + (*n*-5), through 2012); MaxTempSumm (▲) and AvMinTempDur (■). Since recapture probability was modelled as additive for sex, the graph for female recapture probability is similar but slightly less (≈ 4%) at each capture event.

Recapture probability was modelled as covarying (Table 1 and S1 Table) with weather conditions prior to (MaxTempSumm) and during (AvMinTempDur) annual trapping and additively on sex (species and treatment groups were not supported by AIC_c_ selection). The additive structure made male recapture slightly larger than female recapture (≈ 4%) for a given capture event. While both regression coefficients were positive, the standardized coefficient of MaxTempSumm was about 5 times larger than that of AvMinTempDur. Male recapture probability varied from 0.2–0.51 across the study, being higher after warm summers (MaxTempSumm) with a subtle negative influence of cold nights when trapping (AvMinTempDur) (Fig 2).

### Variation in resident survival

**Fig 3:**
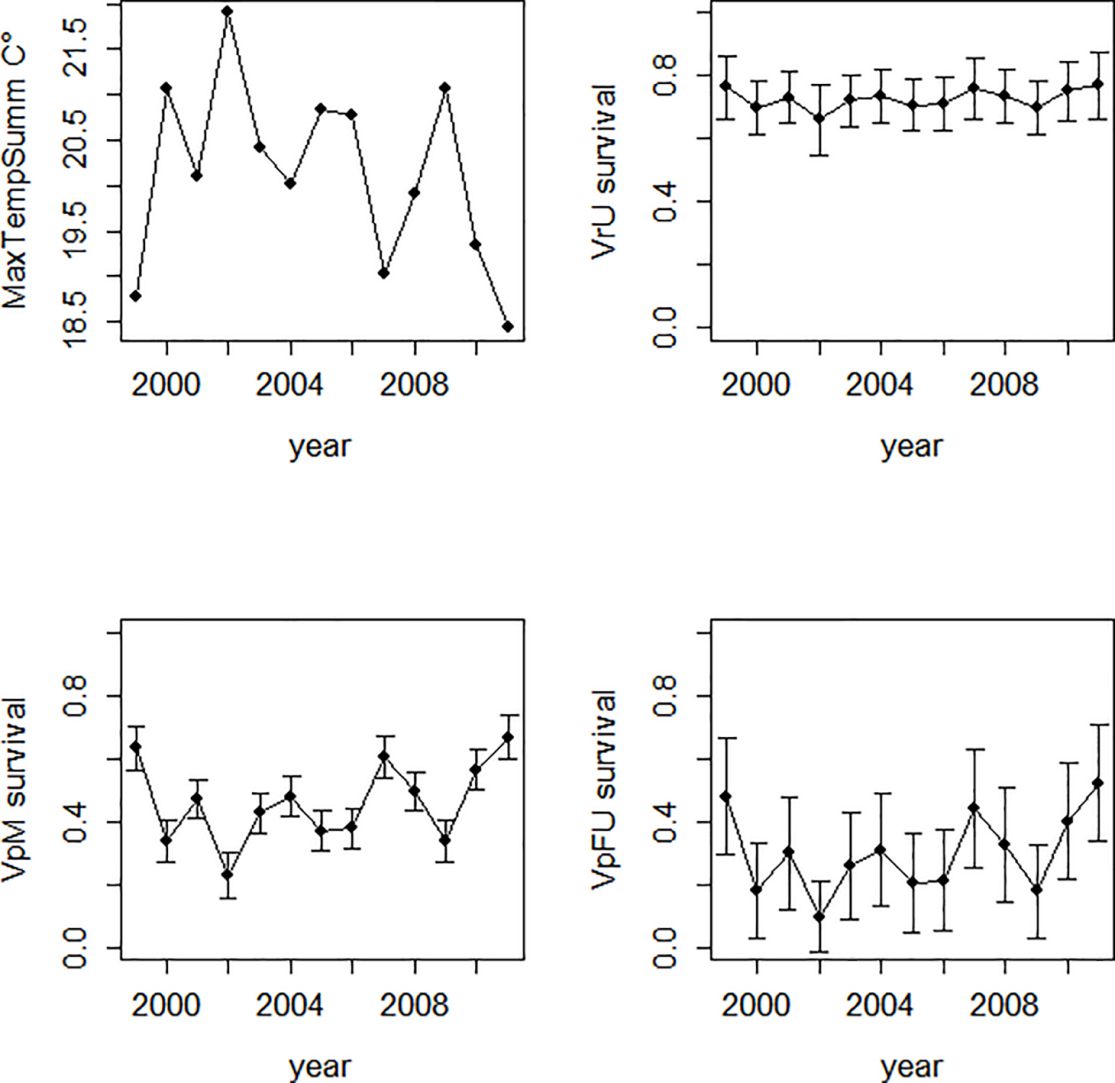
Time varying survival parameters and their covariate (MaxTempSumm). The value plotted over year *n (n* = 1999–2011) is survival over the year beginning with the capture event of year *n* to year *n*+1. VrU = *V*. *regulus* in unlogged; VpM = *V*. *pumilus* males; VpFU = *V*. *pumilus* females in unlogged. Note that survival values are averages over modelled estimates assuming covariation with the weather covariates.

A single, effect-independent survival of 0.89 ±0.04 per two weeks was estimated for survival of already-marked bats between capture events *within* the same year (applicable only to the period of capture). All resident survival rates from the (final) capture event of one year to the (initial) capture event of the next year are expressed as annual rates. Species, sex and treatment effects influenced survival. In line with our prediction that the forest refuge would buffer survival from climate extremes, modelling assigned the species *C*. *morio* and *V*. *darlingtoni*, and also *V*. *regulus* in regrowth, a common constant annual survival rate estimated at 0.60 ±0.02 while for *V*. *pumilus* females in regrowth, annual survival was estimated to be 0.41 ±0.12. The annual survival rates for male *V*. *pumilus*, female *V*. *pumilus* in unlogged, and *V*. *regulus* in unlogged covaried inversely with MaxTempSumm, with the same regression coefficient, differing only in intercept (means ± SD were 0.46 ± 0.13, 0.30 ± 0.12, and 0.73 ± 0.03, respectively; see also Fig 3). Yet variation with MaxTempSumm was small for *V*. *regulus* in unlogged, and only substantial for *V*. *pumilus*, which experienced low survival throughout the study (typically < 0.5). Logging treatment was influential on just two cases of survival, being higher in unlogged than regrowth catchments for *V*. *regulus* (mean survival = ~0.73 vs 0.60), while the reverse was the case for *V*. *pumilus* females (mean = 0.30 vs ~0.41).

### Mean life expectancy and longevity

Mean life expectancy for resident bats that were > 3 months in age at banding was just 1–3 years; *C*. *morio* = 2.0 years, *V*. *darlingtoni* = 2.0 years, *V*. *regulus* = 2.0–3.2 years and *V*. *pumilus* = 0.8–1.3 years. This is considerably less than the maximum longevity we recorded; *C*. *morio* = 8^+^ years, *V*. *darlingtoni* = 9^+^ years, *V*. *regulus* = 8^+^ years and *V*. *pumilus* = 6^+^ years. Although use of apparent survival will underestimate life expectancy (though we accounted for the effect of transients), the extent of teeth wear in the population provides supporting evidence for low mean life expectancy. Across all bats (excluding retraps), 85% showed no signs of teeth wear (index = 1) and just 1% of bats had very worn teeth (index = 3; 6% of retrapped bats), confirming that few bats, even those not banded, live to an old age.

### Variation in abundance

The proportion of residents amongst newly marked bats was computed as an intermediate step in the estimation of the abundance of residents (Supplement). Combining the sexes, *V*. *darlingtoni* was the most abundant species at high and mid altitudes, with *V*. *pumilus* most abundant at low altitude (S3 Table). Elevation was negatively related to abundance of *V*. *pumilus* (r = -0.99; P<0.001) and positively related to the abundance of *V*. *darlingtoni* (r = 0.93; P<0.01) and *V*. *regulus* (r = 0.83; P<0.05), but not significant for *C*. *morio* abundance (r = 0.522; P = 0.29). As a consequence, *V*. *pumilus* was negatively associated with *V*. *darlingtoni* (r = -0.88) and *V*. *regulus* (r = -0.78). The abundance of *V*. *darlingtoni* and *V*. *regulus* were positively associated with each other (r = 0.95). These patterns occurred in both treatments. After adjusting for elevation, pre-planned contrasts found no significant difference in abundance averaged across 14 years between unlogged and regrowth catchments for any species: *V*. *regulus* (*t* = 0.41; P = 0.71), *V*. *pumilus* (*t* = 1.76, P = 0.12), *V*. *darlingtoni* (*t* = 0.06, P = 0.96) and *C*. *morio* (*t* = -0.30, P = 0.89).

**Fig 4:**
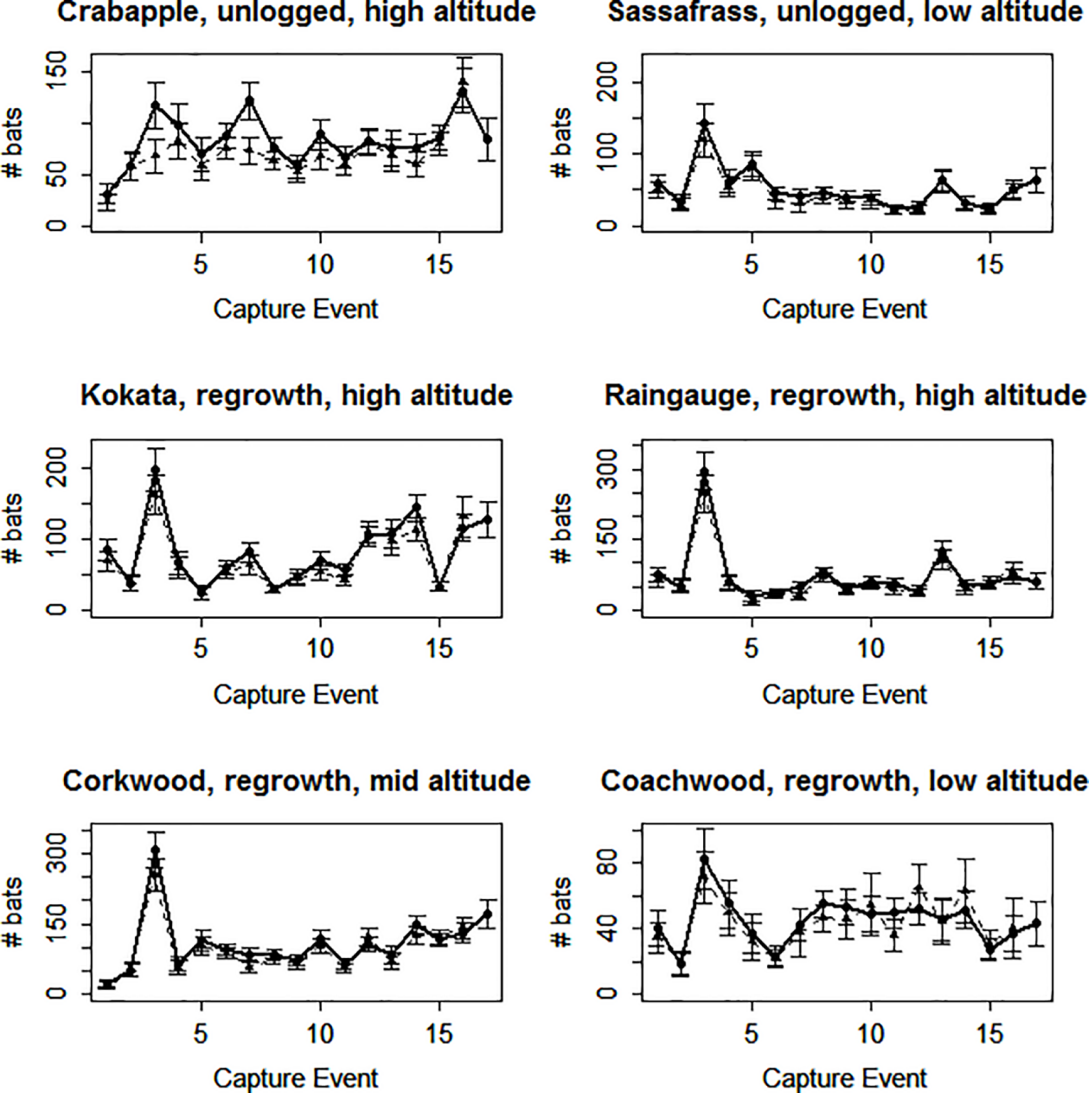
Plots of estimated abundance (●) and number of residents (▲) over all four species per site with respect to capture event, numbered as in Fig 3.

We predicted that abundance would be relatively insensitive to weather extremes over time. The DFA indicated that the time series of abundances behaved randomly and similarly across sites and across species (see Supplement). We attribute the peak in abundance in the 4^th^ capture (Fig 4), early February 2000, to high numbers of juveniles (39% of captures in 2000) still present during the only capture event to take place so early in the year. Removing abundance values for February 2000 did not alter DFA patterns. One site (Kokata) showed a major dip in abundance in the 15^th^ sampling event (2010), while an adjacent catchment (Coachwood) showed a less distinct dip (Fig 4), coinciding with silvicultural thinning of the dense forest at Kokata in 2009–2010, including surrounding trap locations. Abundance recovered in the following year (Fig 4).

### Body condition

Body condition for each species/sex fluctuated only slightly over time (CVs: 0.03–0.08). The DFA analysis detected no evidence for any trend with covariates and only the simplest random walk structure, which again is consistent with our prediction that the forest refuge buffered against weather extremes. Logging treatment did not influence the body condition of residents (P = 0.48), nor was there a significant logging by species interaction (P = 0.29) or logging by species by sex interaction (P = 0.22). Condition was not correlated with bat age (time elapsed since banding; r = 0.17).

## Discussion

Forest bats are considered to be an indicator of ecosystem health [12]. Our study in a long-term experimental forest provides the first comparison of survival rates and abundance of bats between different forest management treatments. The effect of logging history was minor, though variable, for four tree-hollow roosting vespertilionid bats, suggesting regrowth forest is not acting as a sink for these bats [8]. This contrasts with two other studies where bat survival was sensitive to environmental factors, including environmental contamination of a river [19] and fluctuations in the abundance of introduced mammalian predators [21]. There was also no detectable effect of logging history on the abundance or body condition of any of these species.

The long time series also provided an opportunity to explore the relationship between weather extremes and survival, which has been highlighted as a major knowledge gap for assessing impacts of climate change [2, 49]. We found that apparent survival was not strongly influenced by extreme weather patterns, except for the smallest species, nor was abundance or body condition. These results suggest the location of our study area acted as a climate refuge, buffering bat population dynamics from weather extremes and is consistent with the proposed value of climate refuges in mitigating the projected effects of climate change. Because the original goal for our study did not anticipate the extreme drought, we lacked dedicated sites outside our study area to serve as climate refuge controls. While this clearly limits the strength of our conclusions, population dynamic time series at multiple sites are logistically difficult and especially rare [50].

Accordingly, our assessment of the buffering capacity of the climate refuge should be treated with caution, though a number of additional factors add support to our interpretation. First, rainfall recorded at our study site during the extended drought between 2002 and 2007, averaged 190 mm more rainfall per year than the nearest (9 km WSW) weather station at a lower altitude (Chichester Dam, 194 m A.S.L., Australian Bureau of Meteorology). Second, other studies attributed negative impacts of this severe drought on wildlife in other locations in southeastern Australia [32, 33, 34]. Third, survival of little brown bats *Myotis lucifugus* in North America peaked in years with wet summers, and drier summers associated with global climate change are predicted to be a future risk for the species [3].

### Bat population dynamics

From the highly complex global model with time*species*sex*treatment dependence, we derived relatively simple models (at most 20 parameters) whose model-averaged parameters nevertheless portray a relatively complex picture of variation in survival. We caution that more data might paint an even more subtle picture of the dynamics of these four species than our data could reveal. For example, elevation could be an additional influence on survival that we chose not to model in preference to other factors given the number of sites sampled and bats caught per site. We purposely minimised trapping effort to minimise trap avoidance, yet still achieved a 32% retrap rate. It is also important to point out that we estimated apparent survival, as mortality and permanent emigration are confounded. However, by distinguishing between residents and transients, our modelling does not ascribe all disappearances of bats caught only once to apparent mortality of residents, which is therefore a less biased estimate of actual mortality than it would be otherwise. Transience is suspected to be greatest in autumn when juveniles disperse [17]. Our estimates of the proportion of residents (S1 Appendix and S1 Fig) was high (82–90%, with the exception of *V*. *regulus* in unlogged) among trap events *within* autumn, suggesting minor transiency over a 2–3 month period. These results are consistent with the fact that small vespertilionids typically have stable short-term foraging ranges [51], though these can shift locally in relation to patch dynamics of food [52]. Residency *among* years was also high, on average, but it varied considerably across the study (S1 Appendix and S1 Fig; see below). Just one retrap was recorded beyond the immediate surrounds of our study area, which was an 88 km straight line movement for *F*. *tasmaniensis* [53], but this was for the largest species that we banded and such movement is unlikely to be undertaken by smaller species.

Despite these uncertainties, our results are broadly consistent with previous studies on bat survival. First, our survival estimates averaged 0.60–0.73 per annum, except for the smallest species, *V*. *pumilus*, which averaged 0.30–0.41. These estimates (except *V*. *pumilus*) are relatively high compared to similar-sized mammals, but comparable to other bats, whose survival tends to vary between 0.6–0.9 per annum [3, 15, 17, 18, 20, 21, 22, 38]. Although we included 3–4 month old bats in our models, and it is well known that juveniles have elevated mortality and dispersal [3, 17, 21], we found a single, effect-independent survival parameter of 0.891 ±0.035 per two weeks of resident bats during the capture periods of 1999 and 2000. This survival rate is only applicable to the capture period and should not be extrapolated beyond it. An attrition rate of some 11% per two weeks would not be sustainable over many consecutive fortnights, but may reflect apparent mortality, including ongoing dispersal (even accounting for transients), of the cohort of sub-adults during the two extended autumn periods of capture. Pooling of sub-adults (often unrecognisable) with adults likely negatively biases apparent survival. We found no evidence for a sex effect on survival *over* years for the three larger species, but a small positive effect was present for males of the smallest species (*V*. *pumilus*). Differential survival between the sexes of bats has been found in some studies [15, 21], but not others [17].

Our smallest species, *V*. *pumilus*, presents the greatest anomaly in our results, but we suggest there are a number of explanations for lower survival at our study site. First, the species is small (~ 4 g) and is known to give birth to twins [54], both of which are traits related to lower survival [15]. Second, *V*. *pumilus* is a species of the coastal forests of NSW and the higher elevation of our study area is likely to be at the distributional limits of the species. Survival was lower for female *V*. *pumilus*, perhaps suggesting limited resources for breeding. Notably, it was the only species whose abundance was correlated negatively with elevation in our study area, which is also consistent with activity levels measured by acoustic detectors [25]. We suggest that our high elevation study area may be operating as a sink [8] at the edge of the range for *V*. *pumilus*, while negative associations with co-occurring species could imply additional negative effects from competition.

### Population dynamics in disturbed forests

Long-term changes in population dynamics have been described for bird communities in relation to changes in vegetation structure in regenerating forest and responses tend to be species idiosyncratic, though survival estimates are rare [55, 56]. Results from our long-term bat banding found that survival and abundance showed minor differences at best between regrowth (varying in age over the study from 16–30 years) compared to unlogged catchments. There was no detectable effect of logging history on survival of two species (*C*. *morio* and *V*. *darlingtoni*), a positive effect for one (*V*. *pumilus*) and negative for the other (*V*. *regulus*), while there was no detectable effect of logging history on the abundance or body condition of any of these species. All four of the bat species we investigated can be classified as edge-space species with medium to high frequency echolocation calls that commonly avoid clutter, such as in dense regrowth [25]. Radio-tracking also supports our results in that *V*. *pumilus* will roost and breed in regrowth forests [51], while *V*. *regulus* preferentially roosts in patches of mature forest [57]. Acoustic detectors revealed that *V*. *regulus* was more active off tracks in unlogged forest than in regrowth [25], suggesting either that it is more sensitive to clutter than *V*. *pumilus* or it displaces the latter from these areas.

We suggest that survival was not consistently lower in cluttered regrowth forests because tracks provided open linear flyways where bats could forage efficiently. A number of studies have suggested that tracks facilitate the use of regrowth forest by many bat species that would otherwise be too cluttered for efficient foraging by echolocation [25, 27, 28, 29]. However, as tracks only represent ~ 3% of the landscape in timber production forests (e.g. [28]), local availability of unlogged forest is also likely to be important. Unlogged forest was retained within informal reserves along streams to protect this environment and as rainforest because this forest type is no longer logged in NSW [58]. Systematic landscape protection of unlogged forest is a key recommendation for ecologically sustainable management of timber production forests and is often referred to as multi-scale forest management [16, 59]. We note that our results pertain to areas of multi-scale forest management where ecological sustainability is a goal. Also, our conclusions focus on the most abundant local species rather than rare or threatened bat species, which we were unable to band in sufficient numbers for analysis.

The regrowth forest substantially self-thinned over the course of our study coincident with drought, even though rainfall was higher than areas outside of our study, with reduced stem density of both overstorey and understorey species being evident by 2011 [60]. This partly corresponds to trends of increased abundance over the study at two of the four regrowth sites where self-thinning was most evident (Corkwood and Kokata; authors pers. obs.). We also detected a dip in abundance at Kokata in 2010, coinciding with silvicultural thinning at that site. Recovery in the following year suggests this was a behavioural rather than a population effect. Such a pattern may represent lower trap success on trails during thinning, due to bats avoiding the disturbance.

### Variation in population dynamics in relation to weather extremes

Can regenerating forest function as a climate refuge during climate extremes? A key result from our study was little variation in survival over the 14 years of banding, except for *V*. *pumilus* and to a smaller extent for *V*. *regulus*, our two smallest species. Survival rates for both of *V*. *pumilus* and *V*. *regulus* recovered as soon as weather conditions improved. Body condition also showed little variation over the study. This was despite the study spanning a severe El Niño period that resulted in the worst drought in recorded history for south-eastern Australia as well as spanning high rainfall years of La Niña periods. Not only were our species resilient to severe drought, variation in adult survival with extreme weather conditions has been suggested to be a major driver of population dynamics in other bat species and temperate passerines [49]. For example, survival of little brown bats *Myotis lucifugus* peaked in years with wet summers, suggesting drier summers associated with global climate change could pose future risks for the species [3], although the inverse was found for ghost bats *Macroderma gigas* in Australia [23]. European studies have either reported near constant survival in bats over years [18] or annual fluctuations only weakly related to weather [22], though they have not spanned extreme droughts. We suggest that the location of our study site in a climate refuge buffered survival, body condition and abundance from the effects of weather extremes. Annual rainfall at our wet site remained relatively high even in the driest year of 2002 (877 mm). These results are similar to tropical cloud forests where relatively constant capture rates of bats have been reported over a 27 year period [61]. Floodplains are another relatively moist environment that represented a climate refuge for birds during the Australian millennium drought, with an analysis of reporting rates rather than population dynamics finding that fewer species declined in floodplains (19%) than non-floodplains (29%) [62]. We predict that bat survival in Australia is likely to decline outside of such climate refugia during droughts, given canopy invertebrates decline during droughts [63], and collapses were observed in other fauna groups [23, 33, 34]. Climate change is forecasted to be widespread in the forests of eastern Australia [64] and forests may become increasingly vulnerable to tree mortality in response to future warming and drought [65].

There is some evidence that weather conditions had more subtle effects on the bat populations. Hot and dry spring-summer conditions immediately prior to autumn trapping were associated with increased capture (recapture) rate. One explanation is that insect abundance was maintained in the forest refuge over the drought and the warmer than average temperatures led to greater activity and a higher trap rate. Weather conditions during trapping were also important with cooler temperatures being associated with lower trap rates, noting that we avoided periods of heavy rain during trapping. In addition, transiency between years (one minus proportion of resident newly marked bats) was higher in the hot and drier years of 2002–2003 and 2009–2010 compared to cooler, wetter years, when transiency was low between years (S1 Appendix and S1 Fig). Male *V*. *pumilus* were an exception to this pattern, possibly indicating greater site fidelity. During drought, residents were possibly over-whelmed by intruders from nearby areas that experienced harsher conditions than at our buffered site, rather than residents leaving in search of better conditions (given survival was mostly unaffected by weather).

As previously noted, our autumn bat trapping prevented us from reliably discriminating juveniles from adults, which prevented estimation of recruitment. Variable weather patterns are known to affect recruitment in bats. In North America, bat reproduction declines during hotter and drier periods, especially during lactation, with more than 50% of females being non-reproductive during the worst drought years [66]. In European bats, juvenile survival (recruitment) across years is relatively constant, though data on pre-weaning mortality is lacking and extreme drought has not been sampled [17, 22]. The population dynamics of other temperate bats are driven by variation in birth-timing, which is delayed in very cold and wet spring and summer weather [67, 68]. We suggest recruitment likely varied in our climate refuge given small fluctuations in abundance across years and mostly constant adult survival rates. Mortality would be most likely post-parturition as we have found > 90% of females are typically pregnant based on trapping in spring at a range of sites and years throughout NSW (see also [3]).

### Conclusion

We found that apparent survival was not strongly influenced by extreme weather patterns, except for the smallest species, nor was abundance or body condition. These results are consistent with the notion that our study area was located in a climate refuge, buffering bat population dynamics from weather extremes. The relative resilience of study populations to the ambient weather pattern had no detectable interaction with logging history, further suggesting that the apparent climate refuge effect was not compromised by the past logging activity [10]. This differs from limited post-drought recovery observed for birds in wetlands, possibly because of very large-scale disturbances to the system, especially altered flooding regimes [63]. Continued refinement of management practices to improve ecological sustainability, following an adaptive management approach, will be fundamental for managing competing needs in identified refugia [69].

## Supporting information

S1 Appendix Additional details on methods and results. (PDF)

S1 Table Basic statistics for the weather covariates employed in modelling. For the survival covariates, there is a measurement for each year 2000 through 2012, corresponding to annual survival rates. For the recapture covariates, there are measurements for each capture event.
(DOCX)

S2 Table The 22 models from which survival and recapture parameters were obtained by model averaging. Models are described in terms of the additional constraints imposed upon the model φ(1)p(sex+ MaxTempSumm + AvMinTempDur). Survival parameters in the second column of Table 2 were set equal by equating the intercepts in the logistic regressions. See Table 2 for abbreviations.
(DOCX)

S3 Table Estimated bat abundance grouped by site and altitude horizontally and by treatment vertically, and then by species and sex vertically. Columns headed ‘All” are the mean overall abundances at each capture event, including transients and residents, while columns headed ‘Res’ are the mean numbers of residents at each capture event. See Table 2 for descriptions of abbreviations.
(DOCX)

S1 Fig Proportion of residents amongst newly marked bats at annual capture events. Transiency can be calculated as one minus the proportion of resident newly marked bats. See Table 2 for abbreviations.
(DOCX)
