# Supplementary material for: Forest bat population dynamics over 14 years at a climate refuge: Effects of timber harvesting and weather extremes
Source: PLoS One. 2018 Feb 14;13(2):e0191471. doi: 10.1371/journal.pone.0191471 (PMC5812568; doi:10.1371/journal.pone.0191471)
Supplement: S1 Appendix — (PDF) [file pone.0191471.s001.pdf]

Forest bat population dynamics over 14 years at a climate refuge: effects of timber harvesting and climate extremes

Bradley Law[1], Mark Chidel and Peter Law

[1]Forest Science Unit, NSW Primary Industries, Locked Bag 5123, Parramatta 2124, NSW Australia.

S1 Appendix. Additional details on Methods and Results.

Methods

Capture Events

There were three distinct capture events in each of 1999 and 2000, the within-year events at most one month apart. We did not pool the within year capture events as a single capture event in case they were informative, e.g., about dispersion. Though the periods from the final capture event in 1999 (2000) to the initial capture event in 2000 (respectively 2001) were slightly less than a year, survival over these periods is reported as an annual rate so as to be comparable to survival between subsequent successive capture events (2001 to 2002, through 2011 to 2012), all of which were one year apart.

U-CARE Goodness of Fit Testing

We followed McDonald et al. (2005:241) for goodness-of-fit testing, using program U-CARE. For the CJS model species*sex*treatment, U-CARE returned: $\chi2$ =145.9, df = 326, p = 1.0000 (tests of: transients: p = 0.02 (2-sided), 0.01 (1-sided); trap: p = 0.63643). Hence, $\chi2$/df provided no evidence for overdispersion. We then investigated CJS models to gain insight into what dependencies of effects our data could support and found that species was influential on survival and time on recapture. Repeating the U-CARE goodness-of-fit testing for the simpler model time*species did not alter conclusions of the U-CARE analysis so we continued use of AICc for model comparison.

Model Averaging

Given the large set of competitive models we model averaged (Burnham and Anderson 2002) the survival and recapture parameters over the 22 models (Table S2) within four AIC_c units of the best model. For survival rates with respect to time steps, other than that used in the MARK modelling and the proportion of residents amongst newly marked bats, we computed these derived parameters from the averages of the MARK output over models. We refer to these parameters as the average-model parameters. We took advantage of our study, for the sake of comparison with the average-model parameters, to also compute all these derived parameters for each of the models we were to average over, and then computed their averages over these models, referring to these parameters as model-averaged.

The only notable difference between these two approaches to computing averaged derived parameters was for VpFU parameters. This difference was traced to the fact that in those models in which, for survival of newly marked bats between capture events within years, VpFU survival was set equal to Cm=Vd=Vr survival, the survival estimate for newly marked VpFU was biased high as the proportion of residents comes out to be larger than one. These models therefore severely affect the resulting average over models, yielding a higher

proportion of residents than one gets from the average model. This example indicates the advantage of using the average model as such biases are more likely to be averaged out first, before they can influence too much the derived parameter.

Results

Bat species captures and banding

The following species were banded – 256 chocolate wattled bat *Chalinolobus morio* (98 retraps), 763 large forest bat *Vespadelus darlingtoni* (463 retraps), 258 southern forest bat *Vespadelus regulus* (201 retraps), 347 eastern forest bat *Vespadelus pumilus* (173 retraps), 149 Gould's long-eared bat *Nyctophilus gouldi* (13 retraps), 101 eastern falsistrelle *Falsistrellus tasmaniensis* (11 retraps), 17 Golden-tipped bat *Kerivoula papuensis* (0 retraps) and 12 greater broad-nosed bat *Scoteanax rueppellii* (0 retraps).

Expanded Description of Table 2

We used the two-letter species abbreviation to indicate survival rates for that species, and qualify if the rate is sex specific by adding M or F and treatment specific by adding R(regrowth) or U (unlogged), e.g., CmFU, designates the survival rate for female Cm in unlogged treatments. The absence of a qualifier means the survival rate is independent of that effect. Coincidences between such survival rates are expressed with equality signs. With this notation, the φ(1) structure was as follows: For newly-marked-bat survival between successive capture events within years (NwY), Cm=Vd=Vp = VrR (i.e., the survival rates for Cm, Vd, Vp, and Vr in regrowth coincided, i.e., were pooled in the MARK model structure) while VrU (Vr in unlogged) was distinct. Both these rates were modelled as time independent. For already-marked-bat survival between successive capture events within years (AwY), Cm=Vd=Vp=Vr was time independent, i.e., all four species were pooled as a single group with no sex or treatment dependence nor time dependence. For newly-marked-bat-survival between successive capture events in different years (NbY), VpM was time independent (i.e., Vp males, no treatment effect dependence), each of Cm = Vd = VrR (i.e., Cm, Vd, and VrR coincided), VpFU, VrU had temporal dependence through the covariate MaxTempSumm, with the same regression coefficient but different intercepts, while VpFR had temporal dependence through the covariates MinTempWint and 6laggedRain. For already-marked-bat survival between successive capture events in different years (AbY), Cm=Vd=VrR and VpFR were time independent, while each of VpM, VpFU, and VrU had temporal dependence through the covariate MaxTempSumm, with the same regression coefficient as for NbY but their own intercepts (i.e., all the logistic regressions of survival parameters on MaxTempSumm for both NbY and AbY survival parameters have the same additive model, with a single regression coefficient and distinguished only be the intercept).

The Proportion of Residents Amongst Newly Marked Bats

Residency was independent of species, sex, and treatment between trap events *within* our first two autumns. The proportion of residents for newly marked Cm=Vd=Vp=VrR (see Table 3 and above for descriptions of acronyms) was estimated to be 0.90 ± 0.07 at the second capture event within 1999 and 0.82 ± 0.12 at all other capture events followed by a capture event within the same year. Residency amongst newly marked VrU was considerably lower (0.41 ± 0.20 and 0.17 ± 0.16, respectively).

The proportion of residents in newly marked bats *among* years was time dependent (Fig S1). All groups (Cm=Vd=VrR, VpM, VpFU, VpFR, VrU) manifested time dependence in the proportion of residents. For Cm=Vd=VrR and VpFR, residency between years was greater than 0.5, falling to its lowest proportion in the drought year of 2002. Variations were driven by the inverse relationship with MaxTempSumm as survival was modelled as time dependent for newly, but not already, marked bats in this group. Residency of newly marked VpM was also typically > 0.5, with a positive relationship to MaxTempSumm, as survival was modelled as time dependent for already marked, but not newly, marked VpM bats. For VpFU and VrU, survival for both newly and already marked bats varied with time, so the variation in the proportion of residents in newly marked bats did not simply reflect the time variation in the covariates involved. The error bars for these groups tended to be greater, reflecting smaller sample sizes. See S1 Fig for the temporal trend in time-varying proportions of residents amongst newly marked bats.

For Cm=Vd=VrR, VpM, and VpFR, some estimates of the proportion of residents amongst newly marked bats exceeded one. This anomaly derived from our strategy of trading bias for precision in model simplification and selection using AIC. For Cm=Vd=VrR and VpFR, survival of already marked bats ended up being modelled as time independent while survival for newly marked bats was modelled as time independent, and conversely for VpM, while for VpFU and VrU both kinds of survival were modelled as time dependent and estimates of the proportion of residents were at most one. For example, with survival of newly marked VpFR bats varying with MaxTempSumm, and survival of already marked VpFR bats modelled as time independent, an estimate of the proportion of residents greater than one at say the 2008 capture event, reflects an underestimate of survival of already marked VpFR bats 07 – 08 due to the modelled time independent estimate or/and an overestimate of survival of already marked VpFR bats 07 – 08. The latter may result from the pooling of groups that was performed in that all groups for which survival varied with MaxTempSumm had the same regression coefficient, i.e., estimates for a particular group are coerced by this ‘pooling’. As noted before, these choices reflected our strategy of trading bias for precision in our estimates.

Estimates of the proportion of residents amongst newly marked bats of a given group could be kept at most one by modelling newly and already marked bats of that group in the same manner, though such models for Cm=Vd=VrR, VpM, and VpFR were not competitive and may have involved parameters MARK treated as non-estimable. Nevertheless, imposing the same trend in survival with time for newly and already marked bats of the same group might seem *a priori* reasonable, in that residents make up a proportion of newly marked bats. But that imposition enforces a particular trend in the proportion of residents amongst newly marked bats. Rather than *a priori* impose the same trend, or lack of trend, on newly and already marked bats of a given group, our strategy as described in Methods was to allow the data to determine these survival rates independently as much as possible and tolerate any resulting biases in the estimates of the proportion of residents amongst newly marked bats. For Cm=Vd=VrR, VpM, and VpFR, estimates of the proportions of residents exceeding one are best regarded as indicating a very high proportion of residents with the amount by which one is exceeded an indication of the bias in the survival estimates from which the proportion is calculated.

Total Abundance

The means over all capture events of overall abundance and residents per species, sex and site are presented in Table S3. Females were more common than males for *V. darlingtoni* at the

high altitude sites Crabapple (unlogged) and Raingauge (regrowth) and the mid altitude site Corkwood (regrowth) but the reverse was true at the two low altitude sites and Kokata (high altitude, regrowth). For *V. pumilus*, males were more common than females at all sites except Raingauge (high altitude, regrowth) where the difference was marginal. *C. morio* females were almost twice as common as *C. morio* males at the mid altitude regrowth site, and *V. regulus* males tended to be more common than females in regrowth, except at low altitude where sample sizes were low.

Dynamic Factor Analysis of Abundance

In a DFA of a vector of time series, in general this vector is expressed as a linear combination of random walks (of number less than the number of time series), a linear combination of covariates, and residuals. The residuals can be modelled as having a general covariance matrix structure, a diagonal matrix structure (no covariances between residuals but the residuals for each time series are not constrained to be equal), or diagonal and equal (which means that for each time series the residual is modelled as being drawn from the same normal distribution). For the global model for our DFAs of abundance, we chose a specific number of random walks, the covariate MaxTempSumm (the most influential covariate on survival), and the general covariance structure for residuals. For each number of random walks less than or equal to the number in the global model, we either included or excluded the covariate, and considered each of the three covariance structures for residuals. AIC_c was used to rank the resulting models. The DFAs of site-specific time series of abundances and of species-specific abundances were each performed for total abundance and for abundance of residents only.

For the DFA of six site-specific time series, we chose four random walks for the global model. For total abundance, the model with lowest AIC_c was that with a single random walk, with slightly different weightings on the random walk per site, and independently and identically distributed (iid) residuals. The next best model, a single random walk with independent but not identical residuals, was 4.51 AIC_c units higher. The only other model within ten AIC_c units of the best model was that with two random walks, with iid residuals, at 7.78 AIC_c units above the best model. The best model with the covariate MaxTempSumm was that with a single random walk, iid residuals, and the covariate, 14.23 AIC_c units higher than the best model. Results for abundance of residents were very similar.

For the DFA of four species-specific time series, we chose two random walks for the global model. As for the site-specific time series, the patterns for total abundance and abundance of residents were very similar for the species-specific time series. Removing the covariate reduces AICc by almost ten in all cases and typically more than 10. The most preferred models are those with one random walk and no covariate. If the residuals are restricted to have no covariances, then the coefficients for the random walk for each time series, i.e., each species, are of the same order of magnitude (all data is normalized in the model). If the residuals are not constrained to have zero covariances, then the coefficients for the random walk are essentially zero and all variation is explained by the residuals. So, the DFA analysis implies that all four species series behave randomly and much the same way, i.e., they either look like a common random walk with the same regression coefficient (order of magnitude) or they look purely like covarying normal residuals.

These results suggest the influence of MaxTempSumm on survival between years of VpM, VpFU, and VrU was subtle and swamped by the insensitivity of survival to this covariate in the other cases.

Body Condition

For this analysis, we proceeded differently. We began with a single random walk no covariates, and the simplest residual covariance structure. We then included one or both of two covariates, MaxTempSumm and 6LaggedRain. Then we allowed the covariance structure of residuals to be diagonal but unequal. We then repeated these comparisons with an additional random walk. AIC_c ranked as the best model that with a single random walk without covariates and with error covariance matrix diagonal with equal variances (this model has the smallest number of parameters of any DFA model) and is more than 20 AIC_c units below the same model but with a single covariate. Allowing unequal variances in the residual errors raises the AIC_c by about 14 while adding a further random walk raises AIC_c by about 8. This analysis therefore detected no evidence for any trend with covariates and only the simplest random walk structure. Coefficients for the random walk per species and sex were all of the same order of magnitude.

References for Supplement

McDonald TL, Amstrup SC, Regehr EV, Manly BFJ (2005) Examples In: *Handbook of capture-Recapture Analysis*, S. C. Amstrup, T. L. McDonald and B. F. J. Manly (eds), Princeton University Press, Princeton, NJ.
