# Supplementary material for: Forest bat population dynamics over 14 years at a climate refuge: Effects of timber harvesting and weather extremes
Source: PLoS One. 2018 Feb 14;13(2):e0191471. doi: 10.1371/journal.pone.0191471 (PMC5812568; doi:10.1371/journal.pone.0191471)
Supplement: S1 Table — For the survival covariates, there is a measurement for each year 2000 through 2012, corresponding to annual survival rates. For the recapture covariates, there are measurements for each capture event. (DOCX) [file pone.0191471.s002.docx]

**Descriptive Statistics for Weather Covariates**

S1Table: basic statistics for the weather covariates employed in modelling. For the survival covariates, there is a measurement for each year 2000 through 2012, corresponding to annual survival rates. For the recapture covariates, there are measurements for each capture event.

| Survival covariates | Mean ±SD | Range |
| --- | --- | --- |
| MaxTempSumm | 20.13 ± 0.98 | [18.43,21.90] |
| MinTempWint | 6.75 ± 0.42 | [6.04,7.47] |
| 6laggedRain | 822 ±181 | [473,1149] |
| Recapture covariates |  |  |
| AvMinTempDur | 12.7 ±2.0 | [7.8,16.4] |
| MaxTempSumm | 19.92 ± 0.96 | [18.44,21.90] |
