# Supplementary material for: Forest bat population dynamics over 14 years at a climate refuge: Effects of timber harvesting and weather extremes
Source: PLoS One. 2018 Feb 14;13(2):e0191471. doi: 10.1371/journal.pone.0191471 (PMC5812568; doi:10.1371/journal.pone.0191471)
Supplement: S2 Table — Models are described in terms of the additional constraints imposed upon the model φ(1)p(sex+ MaxTempSumm + AvMinTempDur). Survival parameters in the second column of Table 2 were set equal by equating the intercepts in the logistic regressions. See Table 2 for abbreviations. (DOCX) [file pone.0191471.s003.docx]

**The 22 Models Averaged Over.**

S2 Table: The 22 models from which survival and recapture parameters were obtained by model averaging. Models are described in terms of the additional constraints imposed upon the model φ(1)p(sex+ MaxTempSumm + AvMinTempDur). Survival parameters in the second column of Table 2 were set equal by equating the intercepts in the logistic regressions. See Expanded Description of Table 2 above for abbreviations.

| Model | ∆AIC_c_ | Deviance | No.  Parameters |
| --- | --- | --- | --- |
| VrU(AbY) constant; Cm=Vd=VrR(NbY) = VrU(NbY). | 0 | 3479.1 | 19 |
| VrU(AbY) constant; Cm=Vd=VrR(NbY) = VrU(NbY); VpM(AbY) = VpFU(AbY); p(sex+ MaxTempSumm). | 0.2 | 3483.4 | 17 |
| VrU(AbY) constant; Cm=Vd=VrR(NbY) = VrU(NbY); VpM(AbY) = VpFU(AbY). | 0.2 | 3481.4 | 18 |
| Cm=Vd=VrR(NbY) = VrU(NbY). | 1.1 | 3480.2 | 19 |
| Cm=Vd=VrR(NbY) = VrU(NbY); p(sex+ MaxTempSumm). | 1.1 | 3482.3 | 18 |
| VrU(AbY) constant. | 1.3 | 3478.4 | 20 |
| VrU(AbY) constant; p(sex+ MaxTempSumm). | 1.3 | 3480.4 | 19 |
| VrU(AbY) constant; VpM(AbY) = VpFU (AbY); p(sex+ MaxTempSumm). | 1.5 | 3482.6 | 18 |
| VrU(AbY) constant; VpM(AbY) = VpFU(AbY). | 1.5 | 3480.7 | 19 |
| Cm=Vd=Vp = VrR (NwY) = Cm=Vd=VVr=Vp(AwY); Cm=Vd=VrR(NbY) = VrU(NbY);VpM(AbY) = VpFU (AbY); p(sex+ MaxTempSumm). | 1.8 | 3487.6 | 16 |
| Cm=Vd=Vp = VrR (NwY) = Cm=Vd=VVr=Vp(AwY); Cm=Vd=VrR(NbY) = VrU(NbY);VpM(AbY) = VpFU (AbY). | 1.9 | 3485.1 | 17 |
| VrU(AbY) constant; Cm=Vd=Vr(AbY). | 2.0 | 3481.1 | 19 |
| VrU(AbY) constant; Cm=Vd=Vr(AbY); p(sex+ MaxTempSumm). | 2.0 | 3483.2 | 18 |
| φ(1)p(sex+ MaxTempSumm). | 2.5 | 3481.5 | 19 |
| φ(1)p(sex+ MaxTempSumm + AvMinTempDur). | 2.5 | 3479.5 | 20 |
| VpM(AbY) = VpFU (AbY); p(sex+ MaxTempSumm). | 2.6 | 3483.7 | 18 |
| VrU(AbY) constant; Cm=Vd=VrR(NbY) = VrU(NbY) = VpFU(NbY). | 2.6 | 3483.7 | 18 |
| VpM(AbY) = VpFU(AbY). | 2.6 | 3481.8 | 19 |
| Cm=Vd=Vp = VrR (NwY) = Cm=Vd=VVr=Vp(AwY); p(sex+ MaxTempSumm). | 2.9 | 3484.0 | 18 |
| Cm=Vd=Vp = VrR (NwY) = Cm=Vd=VVr=Vp(AwY). | 2.9 | 3482.1 | 19 |
| Cm=Vd=VrR(NbY) = VrU(NbY) = VpFU(NbY). | 3.7 | 3484.8 | 18 |
| Cm=Vd=VrR(NbY) = VrU(NbY) = VpFU(NbY); p(sex+ MaxTempSumm). | 3.9 | 3487.1 | 17 |

Given the many slight variations in these models, and their complex descriptions, we note that simple models constructed from our heuristic global model, i.e., models just depending on the combinations of the group factors and time, including the null model, were not at all competitive, being some 50 AIC_c_ units above the models in Table S2.
