# Supplementary material for: Forest bat population dynamics over 14 years at a climate refuge: Effects of timber harvesting and weather extremes
Source: PLoS One. 2018 Feb 14;13(2):e0191471. doi: 10.1371/journal.pone.0191471 (PMC5812568; doi:10.1371/journal.pone.0191471)
Supplement: S3 Table — Columns headed ‘All” are the mean overall abundances at each capture event, including transients and residents, while columns headed ‘Res’ are the mean numbers of residents at each capture event. See Table 2 for descriptions of abbreviations. (DOCX) [file pone.0191471.s004.docx]

S3Table: Estimated bat abundance grouped by site and altitude horizontally and by treatment vertically, and then by species and sex vertically. Columns headed ‘All” are the mean overall abundances at each capture event, including transients and residents, while columns headed ‘Res’ are the mean numbers of residents at each capture event. See Table 3 for descriptions of abbreviations.

|  | Sites: | Crabapple | |  |  |  |  | Sassafrass | |
| --- | --- | --- | --- | --- | --- | --- | --- | --- | --- |
|  |  | High Altitude | | High Altitude | | Mid Altitude | | Low Altitude | |
|  |  | All | Res | All | Res | All | Res | All | Res |
| U | CmM | 5.17±0.89 | 4.78±0.85 |  |  |  |  | 2.73±0.63 | 2.09±0.48 |
| N | CmF | 5.8±1.0 | 5.6±1.0 |  |  |  |  | 4.37±0.89 | 3.80±0.81 |
| L | VdM | 17.7±1.6 | 15.5±1.5 |  |  |  |  | 8.2±1.1 | 7.6±1.1 |
| O | VdF | 26.1±2.3 | 23.0±2.1 |  |  |  |  | 0.85±0.39 | 0.76±0.33 |
| G | VpM | 6.24±0.95 | 6.1±1.0 |  |  |  |  | 19.6±1.9 | 18.6±2.0 |
| G | VpF | 3.61±0.83 | 2.34±0.74 |  |  |  |  | 13.0±1.8 | 9.8±1.8 |
| E | VrM | 9.4±1.2 | 6.16±0.90 |  |  |  |  | 1.66±0.52 | 1.23±0.43 |
| D | VrF | 8.7±1.3 | 5.78±0.98 |  |  |  |  | 1.17±0.48 | 0.65±0.30 |
|  | Sites: | Kokata | | Raingauge | | Corkwood | | Coachwood | |
| R | CmM | 7.3±1.0 | 7.0±1.0 | 4.24±0.75 | 4.19±0.76 | 6.5±1.0 | 5.20±0.87 | 3.05±0.71 | 2.43±0.60 |
| E | CmF | 8.2±1.4 | 7.0±1.3 | 3.30±0.83 | 2.75±0.73 | 12.8±1.6 | 11.2±1.5 | 4.09±0.86 | 3.81±0.81 |
| G | VdM | 28.1±2.2 | 23.5±1.9 | 14.9±1.5 | 13.3±1.4 | 23.5±2.0 | 20.6±1.8 | 9.9±1.2 | 8.5±1.1 |
| R | VdF | 18.4±2.0 | 15.4±1.8 | 29.1±2.7 | 26.0±2.7 | 31.0±2.6 | 26.9±2.4 | 2.08±0.65 | 1.69±0.55 |
| O | VpM | 4.29±0.74 | 4.63±0.92 | 2.10±0.55 | 2.28±0.63 | 7.5±1.1 | 6.9±1.1 | 11.5±1.3 | 12.3±1.6 |
| W | VpF | 2.38±0.72 | 2.4±1.4 | 3.67±0.84 | 3.6±1.3 | 4.90±0.91 | 4.9±1.2 | 10.8±1.4 | 11.5±2.3 |
| T | VrM | 7.8±1.1 | 6.39±0.96 | 9.5±1.3 | 7.7±1.1 | 12.6±1.4 | 10.8±1.2 | 1.05±0.41 | 0.94±0.38 |
| H | VrF | 4.7±1.0 | 3.44±0.81 | 6.4±1.2 | 6.0±1.1 | 9.5±1.5 | 8.2±1.3 | 2.22±0.56 | 2.09±0.53 |
