# Supplementary material for: Forest bat population dynamics over 14 years at a climate refuge: Effects of timber harvesting and weather extremes
Source: PLoS One. 2018 Feb 14;13(2):e0191471. doi: 10.1371/journal.pone.0191471 (PMC5812568; doi:10.1371/journal.pone.0191471)

S1 Fig. The covariate MaxTempSumm (°C) and proportion of residents amongst newly marked bats at annual capture events. Transiency can be calculated as one minus the proportion of resident newly marked bats. See Table 2 for abbreviations.
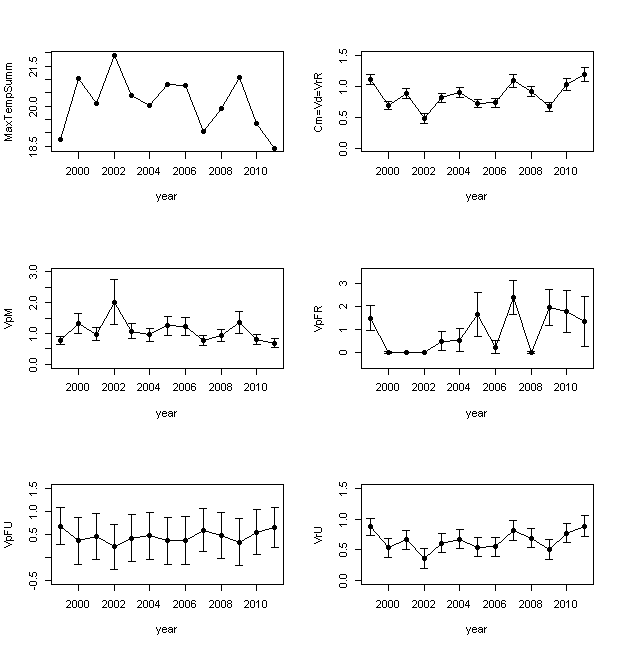

Supplement: S1 Fig — Transiency can be calculated as one minus the proportion of resident newly marked bats. See Table 2 for abbreviations. (DOCX) [file pone.0191471.s005.docx]
